# Aeolus winds impact on volcanic ash early warning systems for aviation

**DOI:** 10.1038/s41598-023-34715-6

**Published:** 2023-05-09

**Authors:** Vassilis Amiridis, Anna Kampouri, Antonis Gkikas, Stergios Misios, Anna Gialitaki, Eleni Marinou, Michael Rennie, Angela Benedetti, Stavros Solomos, Prodromos Zanis, Olympia Vasardani, Konstantinos Eleftheratos, Peristera Paschou, Thanasis Georgiou, Simona Scollo, Lucia Mona, Nikolaos Papagiannopoulos, Christian Retscher, Tommaso Parrinello, Anne Grete Straume

**Affiliations:** 1 Institute for Astronomy, Astrophysics, Space Applications and Remote Sensing (IAASARS), National Observatory of Athens, Athens, Greece; 2 Department of Meteorology and Climatology, School of Geology, Aristotle University of Thessaloniki, Thessaloniki, Greece; 3 Laboratory of Atmospheric Physics, Physics Department, Aristotle University of Thessaloniki, Thessaloniki, Greece; 4 European Centre for Medium-Range Weather Forecasts, Reading, UK; 5 Research Centre for Atmospheric Physics and Climatology, Academy of Athens, Athens, Greece; 6 Department of Geology and Geoenvironment, National and Kapodistrian University of Athens, Athens, Greece; 7 Istituto Nazionale di Geofisica e Vulcanologia, Osservatorio Etneo, Catania, Italy; 8 Consiglio Nazionale delle Ricerche, Istituto di Metodologie per l’Analisi Ambientale, Potenza, Italy; 9 European Space Agency (ESA/ESRIN), Frascati, Italy; 10 European Space Agency (ESA/ESTEC), Noordwijk, The Netherlands

**Keywords:** Environmental impact, Physics

## Abstract

Forecasting volcanic ash atmospheric pathways is of utmost importance for aviation. Volcanic ash can interfere with aircraft navigational instruments and can damage engine parts. Early warning systems, activated after volcanic eruptions can alleviate the impacts on aviation by providing forecasts of the volcanic ash plume dispersion. The quality of these short-term forecasts is subject to the accuracy of the meteorological wind fields used for the initialization of regional models. Here, we use wind profiling data from the first high spectral resolution lidar in space, Aeolus, to examine the impact of measured wind fields on regional NWP and subsequent volcanic ash dispersion forecasts, focusing on the case of Etna’s eruption on March 2021. The results from this case study demonstrate a significant improvement of the volcanic ash simulation when using Aeolus-assimilated meteorological fields, with differences in wind speed reaching up to 8 m/s when compared to the control run. When comparing the volcanic ash forecast profiles with downwind surface-based aerosol lidar observations, the modeled field is consistent with the measurements only when Aeolus winds are assimilated. This result clearly demonstrates the potential of Aeolus and highlights the necessity of future wind profiling satellite missions for improving volcanic ash forecasting and hence aviation safety.

## Introduction

Volcanic ash emitted at aircraft cruising altitudes (9–11 km) constitutes a significant safety and financial risk to aviation^1^. It can cause both temporary aircraft engine failure and permanent engine damage^2^, unreliable readings of critical navigational instruments and poor visibility^3,4^, while it can incur long term economic damage by reducing the service life of the engine, or its components^5^. Hence, during volcanic eruptions, ash-contaminated airspace poses restrictions which disrupt air traffic, leading to large financial losses. The most extreme incident of the Eyjafjallajökull eruption in 2010 grounded more than 100,000 flights from April 15 to 21, causing airlines a cost of about 1.7 billion USD^6^, whereas the global economic damage was estimated at almost 5 billion USD^7^. Before the Eyjafjallajökull eruption, the International Civil Aviation Organization (ICAO) had a zero-tolerance policy, recommending all encounters with ash clouds to be avoided. Current risk management procedures applied by airline companies are more complex due to the need to continue operations in low ash concentration airspace. The information on volcanic ash dispersion after eruption is provided to operators by specialized early warning systems (EWSs) operated by the Volcanic Ash Advisory Centres (VAACs)^8^. These EWSs are commonly based on deterministic atmospheric dispersion and transport models coupled to a global numerical weather prediction (NWP) model, to provide short-term forecasts of volcanic ash cloud^9,10^. As the number of flights in volcanically active regions are expected to increase in the future (e.g., EUROCONTROL Forecast 2020–2028)^11^, likely causing more frequent events of ash encounters, the challenge is to reduce uncertainties in the short-term forecasts of the volcanic ash cloud.

The major sources of uncertainties in the deterministic transport and dispersion models included in the EWSs, are associated with the eruption source parameters (e.g. total mass, plume height), the various model parametrizations (e.g. wet deposition) and the driving meteorology^12,13^. Depending on the injection height at source, which in many EWS is assumed to be a simple function of the mass eruption rate^14^, the long-range transport of volcanic particles is governed by tropospheric and/or stratospheric winds, and particularly the vertical wind shear, which is commonly misrepresented in many NWP models^15,16^. Under the Global Observing System (GOS), wind profile measurements are obtained from radiosondes, commercial aircraft ascents and descents^17^ and ground-based wind lidars and radars. The distribution of these measurements is, however, sparse, and not homogenous, lacking global coverage as most observations are performed over land and mainly in the Northern Hemisphere. In addition, satellite wind information can be retrieved from passive remote sensing imagers, by tracking clouds and areas of water vapor in sequences of satellite images^16^. This approach provides excellent horizontal and temporal coverage, but does not provide sufficient coverage in the vertical dimension^18–20^. Wind is also provided indirectly via satellite-derived mass information (temperature and humidity), and 4D-Var tracer effects for humidity information^21,22^. Additionally, several operational meteorological satellites measure the ocean surface vector wind information by microwave sensing of the ocean surface^23,24^. Although these instruments constrain mesoscale weather features at the ocean surface^25^, they do not have a significant impact on the forecast models in the middle and upper troposphere, where long-range transport of volcanic ash usually takes place^26^. Filling the major gap on wind profiling in the GOS is the highest priority for NWP and climate modeling communities, according to the World Meteorological Organisation (WMO) Rolling Review of Requirements^27^.

This need motivated the Aeolus mission of the European Space Agency, carrying the first high-spectral-resolution Doppler lidar (HSRL) placed in space, dedicated for wind profile measurements^28,29^. Launched in August 2018, Aeolus acquires profiles of the wind on a global scale, filling vast observation gaps particularly over the oceans, poles, tropics, and the Southern Hemisphere, providing homogeneous wind profiling observations. Aeolus mission has already demonstrated a beneficial impact in global NWP models^22^, especially in the tropics. It has been shown that improvements are to be expected also for short-range forecasts of severe weather situations, the analysis of tropical dynamics, and for a better representation of meso-scale circulation systems at midlatitudes^30–33^. Aeolus improves the wind forecast by 0.5–2% in terms of root-mean-square error, an enhancement that is maintained into the medium range. The impact on temperature and humidity has similar patterns. Even the northern hemisphere extratropics geopotential at 500 hPa (~ 5 km) is improved up to day 4 by ~ 0.5–1%. This is despite the widespread presence of high-quality conventional data (radiosondes, aircraft). The largest impact occurs at ~ 100 hPa in the tropics, particularly the east Pacific Ocean^34^. Furthermore, apart from the wind profiles, Aeolus provides aerosol and cloud profiles that could be used for assimilation or evaluation of volcanic ash dispersion modeling. However, these retrievals suffer from the lack of an instrument channel for the detection of the cross-polarized returns. This is a critical part of the backscattered radiation from non-spherical targets such as volcanic ash, and as such, Aeolus should be used with caution in these cases.

Aeolus mission has proved its capacity to improve wind forecasts, especially over under-sampled regions such as the tropics. Similarly, Aeolus can be used over under-sampled remote sites with active volcanoes^22^, towards improving the simulations of volcanic ash dispersion following eruption. In this study, we explore the potential of Aeolus to reduce the uncertainties in volcanic ash forecasting for aviation safety. Based on a system consisting of a regional NWP model that drives a Lagrangian model for simulating ash transport and dispersion, we demonstrate that the Aeolus wind profiles can considerably improve volcanic ash forecasts, particularly beyond the typical + 6 h forecast window provided by many EWS. We analyze the Etna eruption in March 2021, where Aeolus had a close overpass and provided observations around the volcano. Furthermore, the volcanic plume emitted by this eruption was recorded by downwind surface-based lidar measurements at the PANhellenic Geophysical Observatory of PANGEA in the Eastern Mediterranean, allowing direct comparisons of the observations against forecasts (with and without Aeolus assimilation, denoted as “w” and “w/o” Aeolus, hereafter).

## Aeolus impact on the regional meteorological model

**Figure 1:**
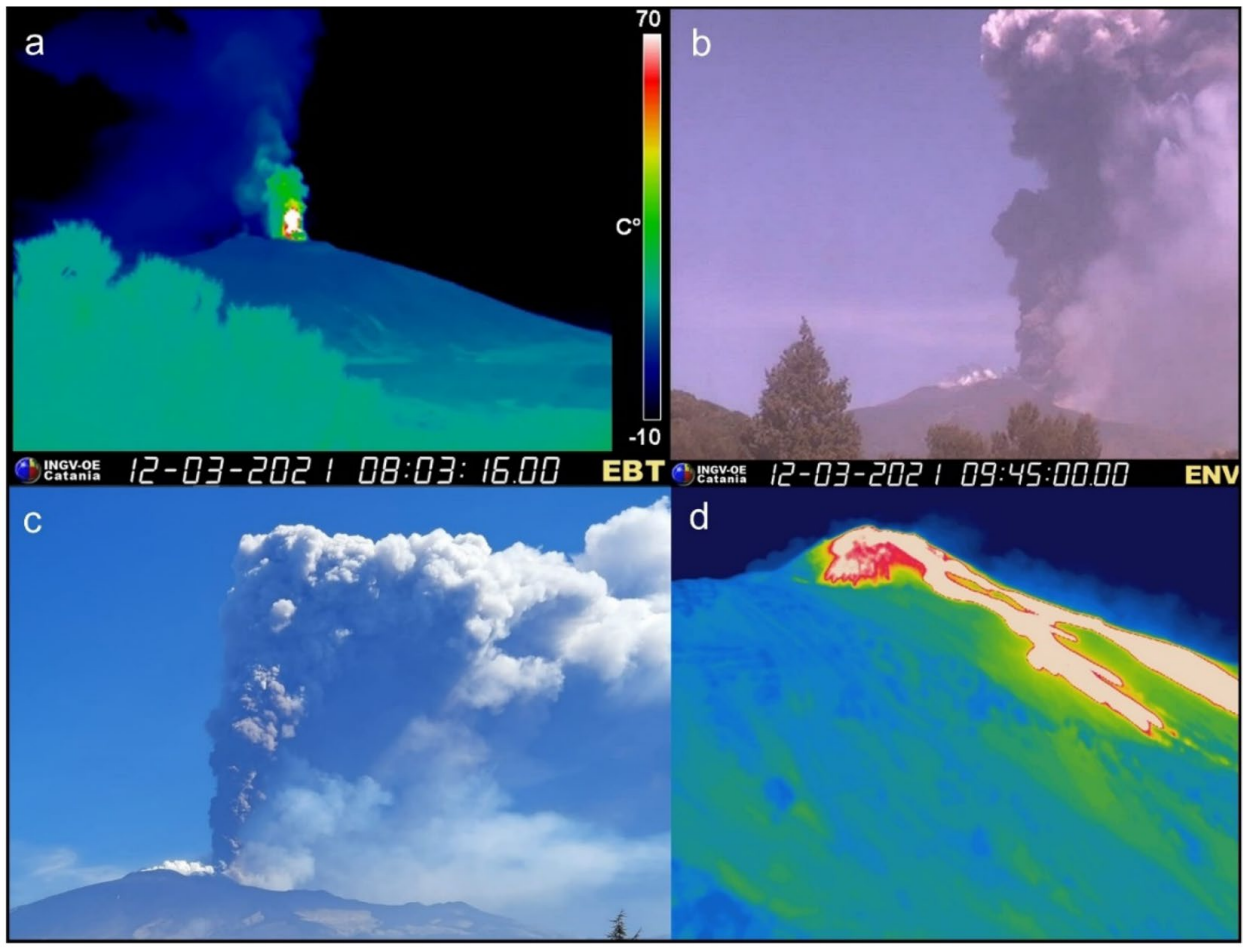
Etna activity on 12th of March 2021, as depicted by the INGV-OE video-surveillance systems and monitoring activities. (**a**) Lava fountain from the South East Crater observed on 12 March 2021 by the thermal camera; (**b**) the eruption column formed during the paroxysmal phase, ranging between 4.0 and 9.5 km, as estimated by the visible camera; (**c**) photo taken by Simona Scollo on the south flank of the volcano during the monitoring activities of INGV-OE; (**d**) lava flow emission retrieved by the thermal camera.

Mt. Etna in Italy is one of the most active volcanoes on Earth. Since February 2021, this stratovolcano experienced several paroxysmal episodes resulting in frequent tephra and sulfate emissions. Here, we focus on the event of the 12th of March 2021, one of the most powerful lava fountain episodes occurred at the South East Crater since 2020^35^. Strombolian type activity began at 02:35 UTC, increasing in frequency and intensity until 07:35 UTC, when the video-surveillance cameras of the Istituto Nazionale di Geofisica e Vulcanologia, Osservatorio Etneo (INGV-OE) depicted the formation of a sustained lava fountain (Fig. 1a). During the paroxysmal phase, the eruptive column gradually increased up to approximately 9.5 km height above sea level (Fig. 1b). The variation of the eruption column was detected by the visible camera calibrated by the INGV-OE^36,37^. The volcanic plume drifted eastwards by the westerly winds (Fig. 1c) that were dominant over the eastern Mediterranean region at the time. Moreover, the activity formed a copious tephra fallout that covered several towns located on the east flank, as well as a lava flow field spreading on the east and north-east flank of the crater (Fig. 1d). The explosive activity and lava flow emission ended at about 10:55 and 12:00 UTC respectively^35^. Here, we focus over the period between 07:15 and 08:44 UTC, on 12 March 2021, when the strongest ash emission was reported by the INGV-OE issue of the Volcano Observatory Notice for Aviation (VONA).

From the field observations near the volcano and the VONA messages, the initial injection height is estimated to 9 km a.s.l. This value is used for the initialization of the volcanic ash dispersion simulations performed with the FLEXPART Lagrangian model. FLEXPART ash transport and dispersion model is driven by wind fields simulated by the WRF regional meteorological model (version 4)^10^, which in turn takes initial and boundary conditions by the ECMWF-IFS global model^38^ (for more information see “Methods” section).

**Figure 2:**
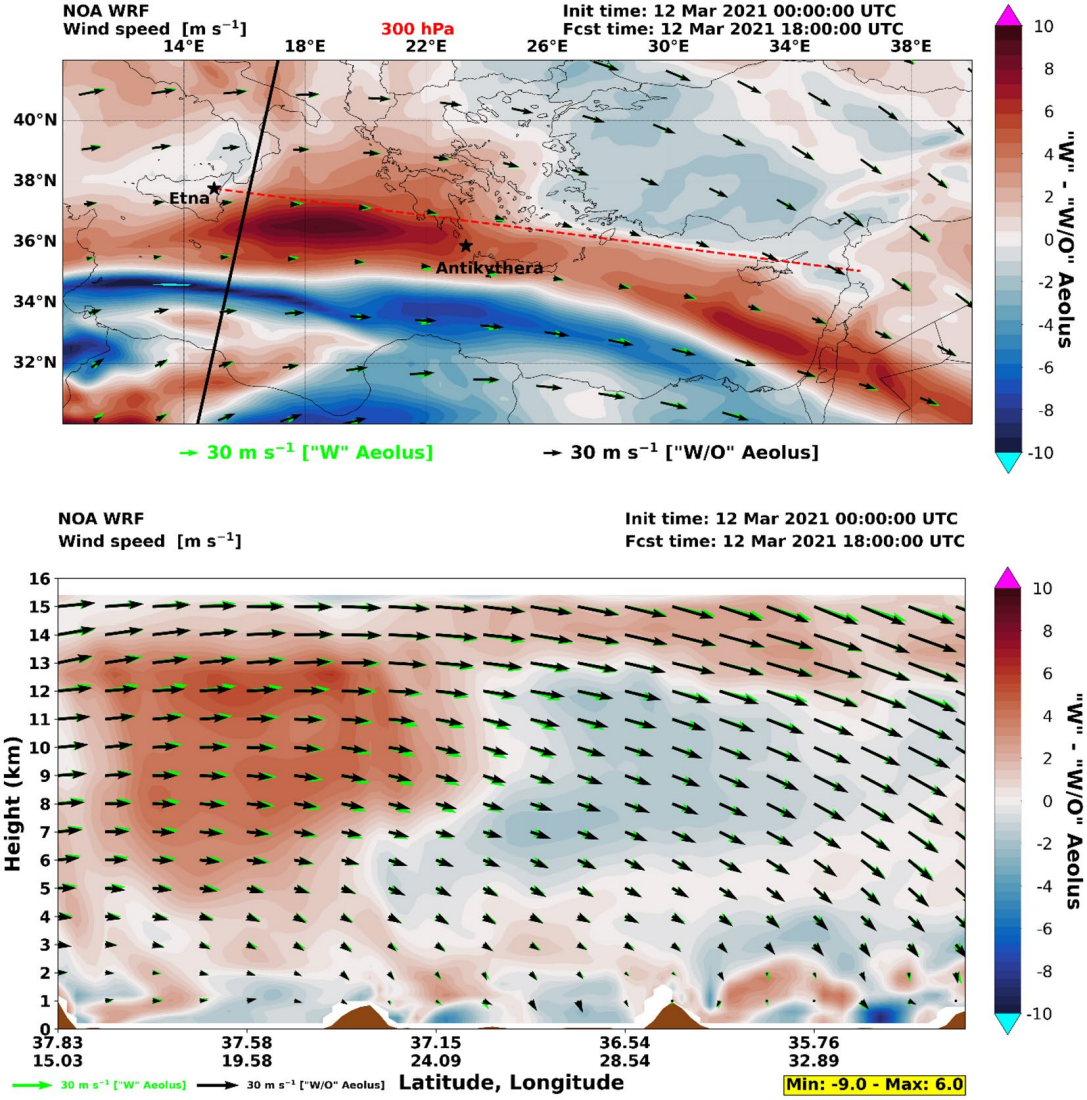
Wind speed differences in WRF 18 h forecasts (“w”–“w/o” Aeolus assimilation). (Upper panel) horizontal winds at 300 hPa (~ 9.6 km); the solid black line indicates the Aeolus orbit path (overpass on 12th of March 2021, 05:01 UTC); (Lower panel) vertical cross-section over the dashed red line denoted in the upper panel (from Etna towards the Middle East); the arrows in both panels are horizontal wind speed components with green denoting “w” Aeolus and black denoting “w/o” Aeolus; the color shading in both panels illustrates the differences in wind speeds between the two WRF runs.

From the inspection of the WRF regional model wind vectors at upper-tropospheric heights (see wind arrows in Fig. 2 at 300 hPa, ~ 9.6 km), it is evident that the general atmospheric circulation at 18:00 UTC (about 11 h after the Etna eruption), remains relatively zonal over the Mediterranean, with westerlies prevailing throughout the troposphere and turn into north westerlies over the Anatolian Plateau and the eastern Mediterranean Sea. This atmospheric pattern favored the direct transport of Etna plumes towards Greece and the Eastern Mediterranean.

To assess the sensitivity of the volcanic ash transport to the driving meteorology, we performed two simulations with the WRF regional model, driven by two versions of the ECMWF-IFS global model, one “w” and one “w/o” Aeolus assimilation. The color shading in Fig. 2 illustrates the differences between the two WRF runs on 12th of March 2021 (18:00 UTC). This comparison indicates significant strengthening of the winds at 300 hPa when Aeolus wind profiles are assimilated (upper panel). Maximum difference values of the order of 8 m/s, accompanied by slight differences in wind vector direction (“w” Aeolus (green) and “w/o” Aeolus (black)) are found over the Ionian Sea (from W to NW) and over the Eastern Mediterranean between Crete and Cyprus (from WNW to NW), where the two jet streams merge.

**Figure 3:**
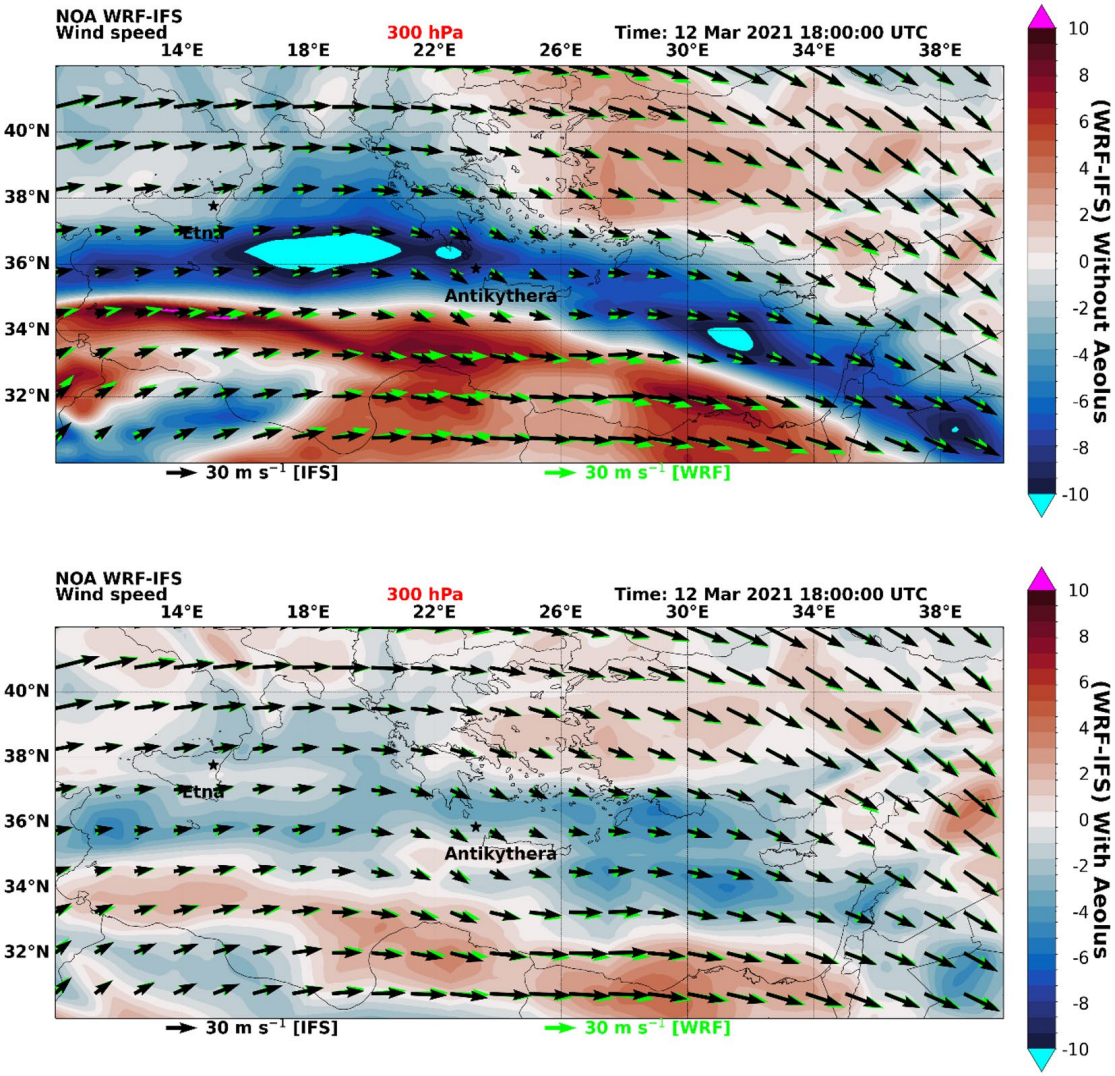
Wind speed differences in WRF 18 h forecasts (“w” and “w/o” Aeolus assimilation in IFS initial state) against the corresponding IFS analyses. Upper panel: Horizontal wind speed differences at 300 hPa (~ 9.6 km) WRF-IFS control run. Lower panel: Horizontal wind speed differences at 300 hPa (~ 9.6 km) WRF-IFS with assimilated Aeolus wind fields; the arrows in both panels are horizontal wind speed components WRF (green) and IFS (black).

To understand those relatively large differences in wind speeds at + 18 h forecasts, we compare the WRF “w” and “w/o” forecast fields against the corresponding IFS analyses (Fig. 3), which serve as reference. As it is clearly seen in the illustration example at 18 UTC on 12th March 2021, (when the plume crossed the island of Antikythera), the WRF-IFS deviations at 300 hPa are significantly suppressed in the “w” experiment in contrast to the “w/o” run. Therefore, a pronounced reduction of the forecast error propagation is revealed, which is attributed to the use of assimilated Aeolus wind profiles, explaining the large differences shown in Fig. 2. Similar tendencies are found at 200 hPa whereas weaker “signals” are recorded in the middle troposphere (500 hPa), weakening further down at ~ 3 km (700 hPa) and diminishing in the lower troposphere. Based on our numerical experiments, the WRF-IFS wind speed deviations are strengthening progressively for increasing lead times in the “w/o” run, whereas an opposite tendency is found in the “w” experiment. This finding justifies the beneficial impact of Aeolus winds assimilation on short-term NWP, which has also previously documented^22^, particularly for the upper troposphere.

The Aeolus assimilation effect in the vertical is also evident for the middle and upper tropospheric layers (mostly between 7 and 15 km), over the west part of the domain that is stronger affected by the Aeolus observations (the black line on Fig. 2, upper panel, denotes the Aeolus overpass). This difference is propagated downwind, where changes of about 2–6 m/s are still evident at jet stream heights over the entire domain. The impact of Aeolus assimilation at these layers is important since both volcanic ash transport and aviation paths are typically located at similar heights. The difference in lower tropospheric winds is also evident, especially when crossing topographic barriers, and additionally at the eastern part of the domain due to the subsidence of middle tropospheric air masses.

## Aeolus impact on the volcanic ash transport

**Figure 4:**
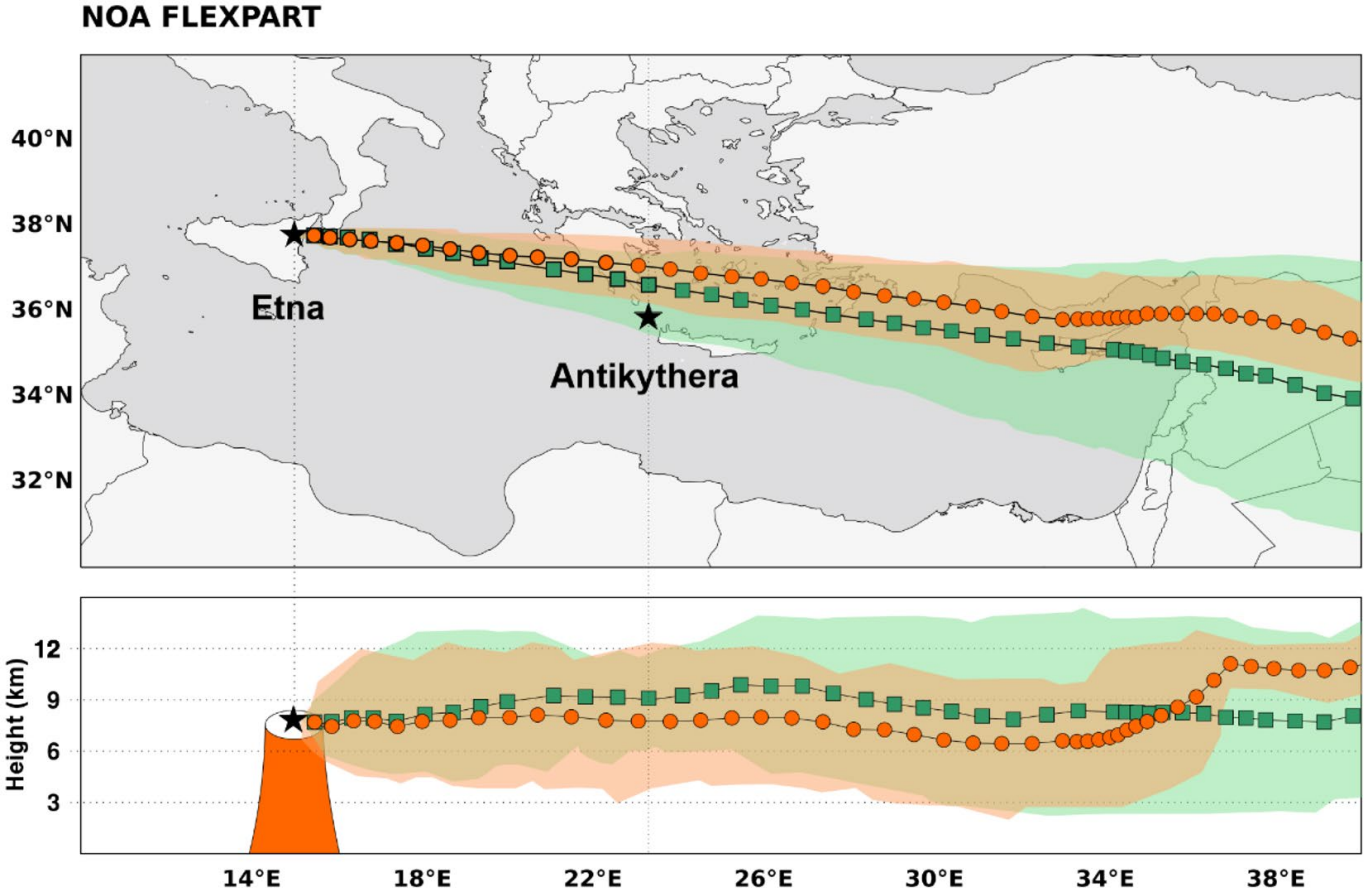
FLEXPART footprint of the volcanic ash plume. Extent of the volcanic plume and footprints of the plume’s center of mass, using meteorological fields “w” Aeolus assimilation (green areas and squares) and “w/o” Aeolus assimilation (orange areas and circles); the period covered is from 12th of March 2021, 07:15 UTC to 14th of March 2021, 00:00 UTC; (upper panel) 2D transport; (lower panel) vertical dimension over the 2D footprints.

To simulate volcanic ash plume transport, we utilize the FLEXPART dispersion model, driven by the wind forecasts produced by the regional model (WRF) instead of the global runs (ECMWF-IFS). This option is preferred because the volcanic plume fluctuations at short time scales can have significant impact on the long-range transport of the ash particles, and such fluctuations cannot be adequately represented by the 6-h ECMWF wind fields. The simulated footprints of the volcanic plume extent and of the plume’s center of mass are shown in Fig. 4 for meteorological fields “w” (green areas and squares) and “w/o” (orange areas and circles) Aeolus data assimilation.

**Figure 5:**
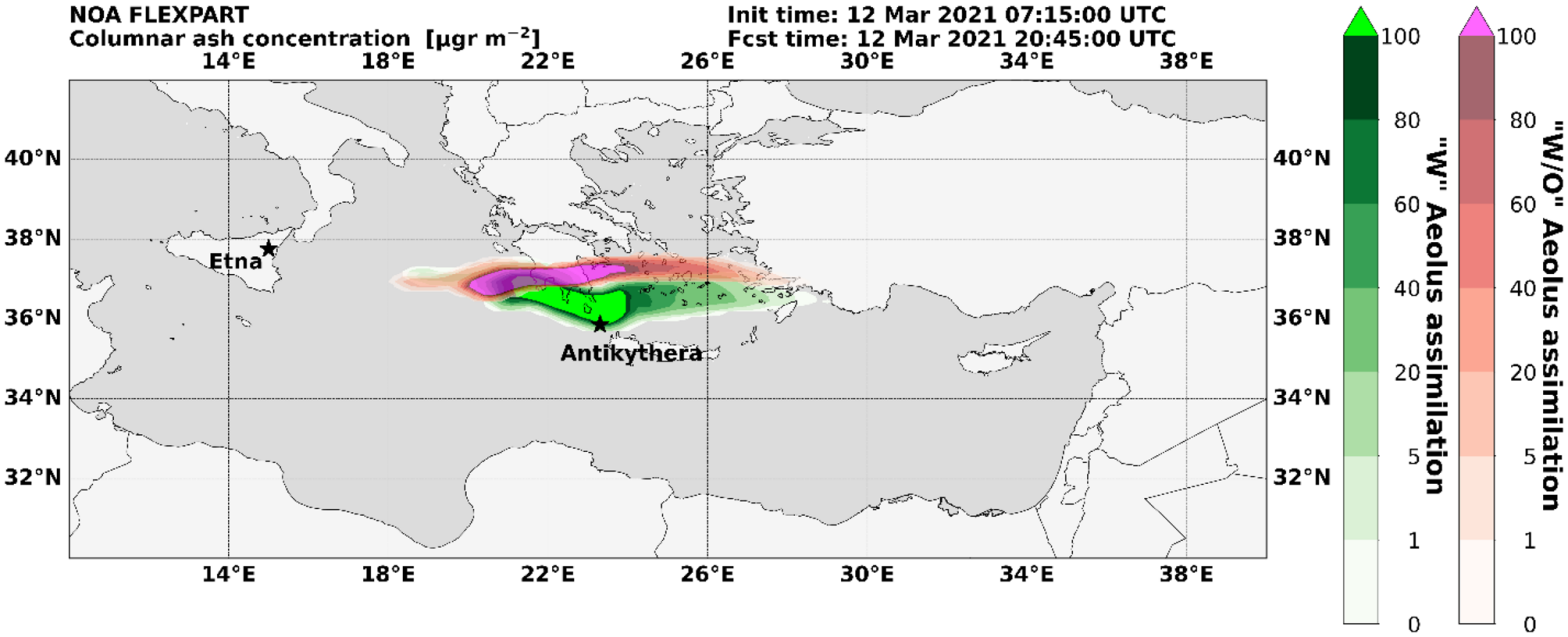
FLEXPART simulations of the volcanic ash dispersion “w” and “w/o” Aeolus assimilation. Ash column loading (μg/m^2^) over the PANGEA observatory in Antikythera, Greece, using meteorological fields “w” (green) and “w/o” (red) Aeolus wind assimilation (12th of March 2021, 20:45 UTC).

The two simulated tracks of the volcanic plume (“w” and “w/o” Aeolus), start to deviate above Greece, due to the wind field differences introduced by the Aeolus assimilation (see also Fig. 2). The volcanic ash plume in the “w” Aeolus forecast expands southwards and reaches Antikythera on 12th of March 2021 at 20:45 UTC (Fig. 5), while the volcanic plume in “w/o” Aeolus forecast never crosses Antikythera, as the forecasted cloud is displaced northward. This provides us a unique opportunity to validate the two forecasts, provided the volcanic ash cloud is measured by the PANGEA observatory in the Antikythera island.

**Figure 6:**
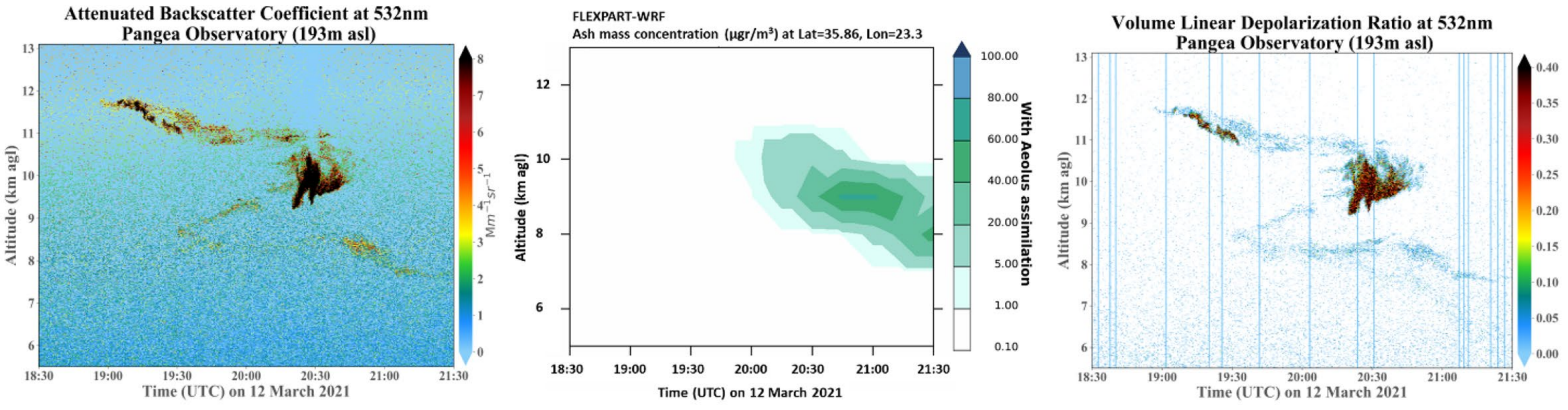
Time-height cross-sections over the PANGEA observatory in Antikythera, Greece. (Left panel) time-height cross-sections of the attenuated backscatter coefficient over Antikythera, Greece; (middle panel) time-height plot of FLEXPART volcanic ash concentrations over Antikythera “w” (green) Aeolus wind assimilation; (right panel) time-height cross-sections of the volume linear depolarization ratio (Polly^XT^-NOA lidar in PANGEA observatory, 12th of March 2021, 18:30–21:30 UTC).

A thorough evaluation of the different plume dispersion simulations is performed against quality-assured aerosol profiling measurements conducted with the Polly^XT^ lidar system^39^, operating at the PANGEA observatory. Figure 6 (left, right), shows the time-height plot of the lidar measurements, depicting a dense aerosol layer in the height range between 7 and 12 km, approximately 11 h after the eruption (18:30–21:30 UTC). The layer is associated with the volcanic ash advection from Etna, since the particle linear depolarization ratios are very high (of the order of 40–50% at the center of the plume), values that are typical for the non-spherical shape of volcanic ash particles^40–42^ (see also Fig. 6, right). The corresponding FLEXPART vertical time-height cross-sections of forecasted volcanic ash concentrations (Fig. 6, middle) show a similar distribution for the observed volcanic aerosol layer over Antikythera, only when Aeolus assimilated fields are used (aerosol layer between 7 and 11 km). On the contrary, the control run (“w/o” Aeolus assimilation) fails to represent the observed structures over Antikythera at all.

**Figure 7:**
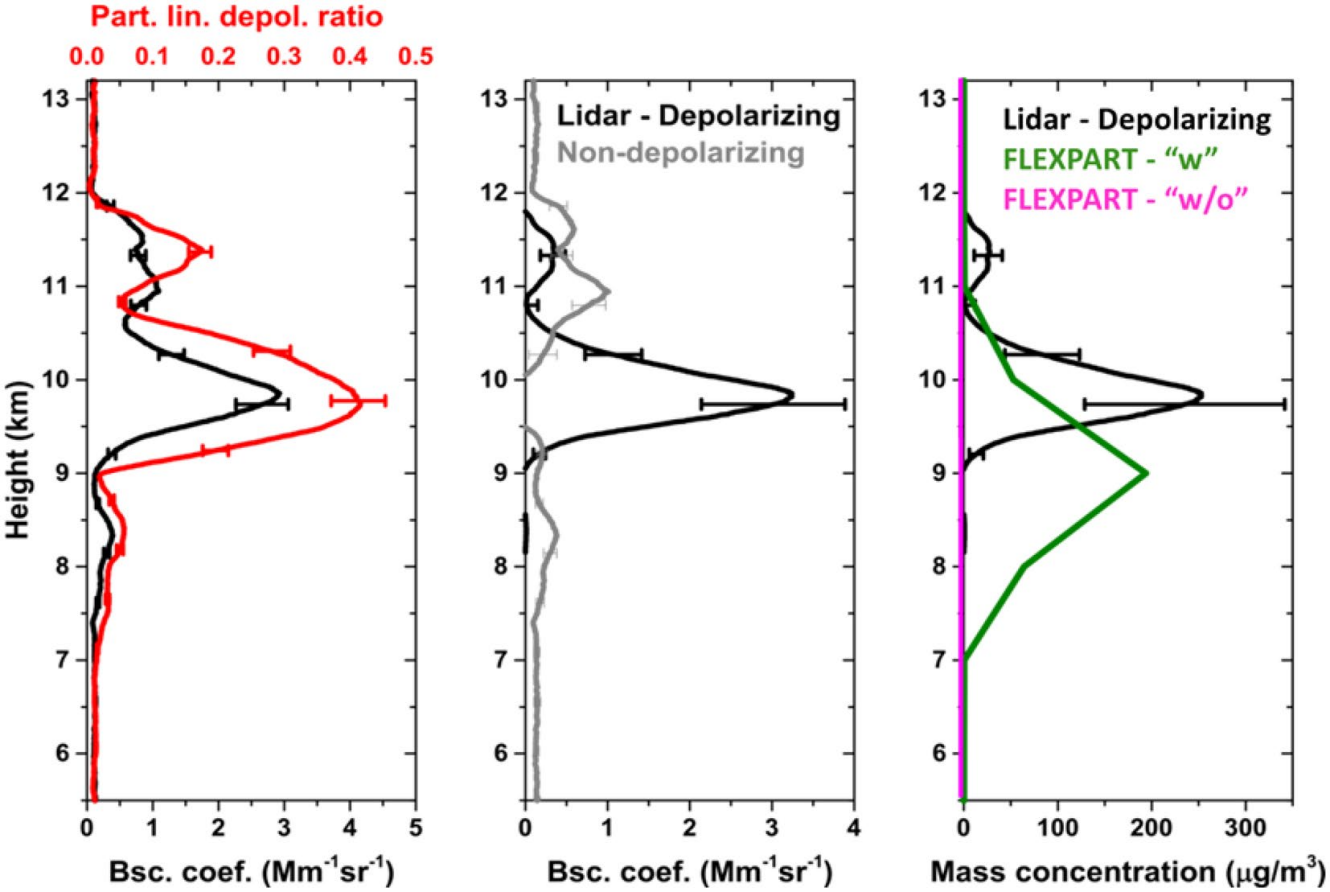
Lidar-derived optical properties and observed/modelled concentration profiles over the PANGEA observatory on 12th of March 2021 (18:30–21:30 UTC). Vertical distributions of: (**a**) total backscatter coefficient (black line) and particle linear depolarization ratio at 532 nm (red line); (**b**) volcanic ash (depolarizing; black line) and sulfate (non-depolarizing; gray line) particle backscatter coefficient; and (**c**) volcanic ash mass concentrations using the POLIPHON lidar method (black line) and from FLEXPART model simulations “w” Aeolus assimilated winds are (green line) and “w/o” Aeolus simulation (red line, equals to zero).

In Fig. 7, lidar profiles of the retrieved aerosol optical properties are additionally shown. We use the POLIPHON method^43,44^ to derive the pure-ash mass concentration profiles. First, we use the particle backscatter and the depolarization ratio profiles (Fig. 7a) as derived from Polly^XT^ observations, and we separate non-spherical from spherical particle contributions to the total backscatter (Fig. 7b). Then, the volcanic ash concentration profiles are retrieved (Fig. 7c) (for more information see the “Methods” section). Our retrievals show that volcanic ash concentrations over PANGEA reached up to almost 250 μg/m^3^ at the plume’s center of mass (black line in Fig. 7c). The POLIPHON method for the estimation of the lidar optical properties and the subsequent conversion to mass concentration, yield an overall uncertainty of ~ 40%^45^ marked with a black error bar in Fig. 7a. The corresponding derived mass concentrations from FLEXPART are also shown in Fig. 7c for comparison (green line). The simulated concentrations agree rather well with the lidar-derived product (and within its uncertainty space). Modelled ash concentrations reached approximately 220 μg/m^3^ at the plume’s center of mass (green line in Fig. 7c) and are in good spatio-temporal agreement with the lidar retrievals despite a slight vertical shift observed between the modeled and observed profiles.

## Air traffic exposure to volcanic ash

The Etna eruption of 12th of March had the potential to disrupt the air traffic in the Eastern Mediterranean. However, it posed a low risk for aviation safety given that the simulated maximum ash concentrations at some distance from the volcano vent did not exceed the ICAO threshold of 2 mg/m^3^^3^. Regardless the low risk for aviation of the specific case study, Aeolus assimilation showed a considerable improvement on the simulated shape and position of the ash cloud (see Fig. 5). These results demonstrate the high impact of Aeolus for aviation safety when the satellite product will be applied in future volcanic eruptions with considerable emissions.

**Figure 8:**
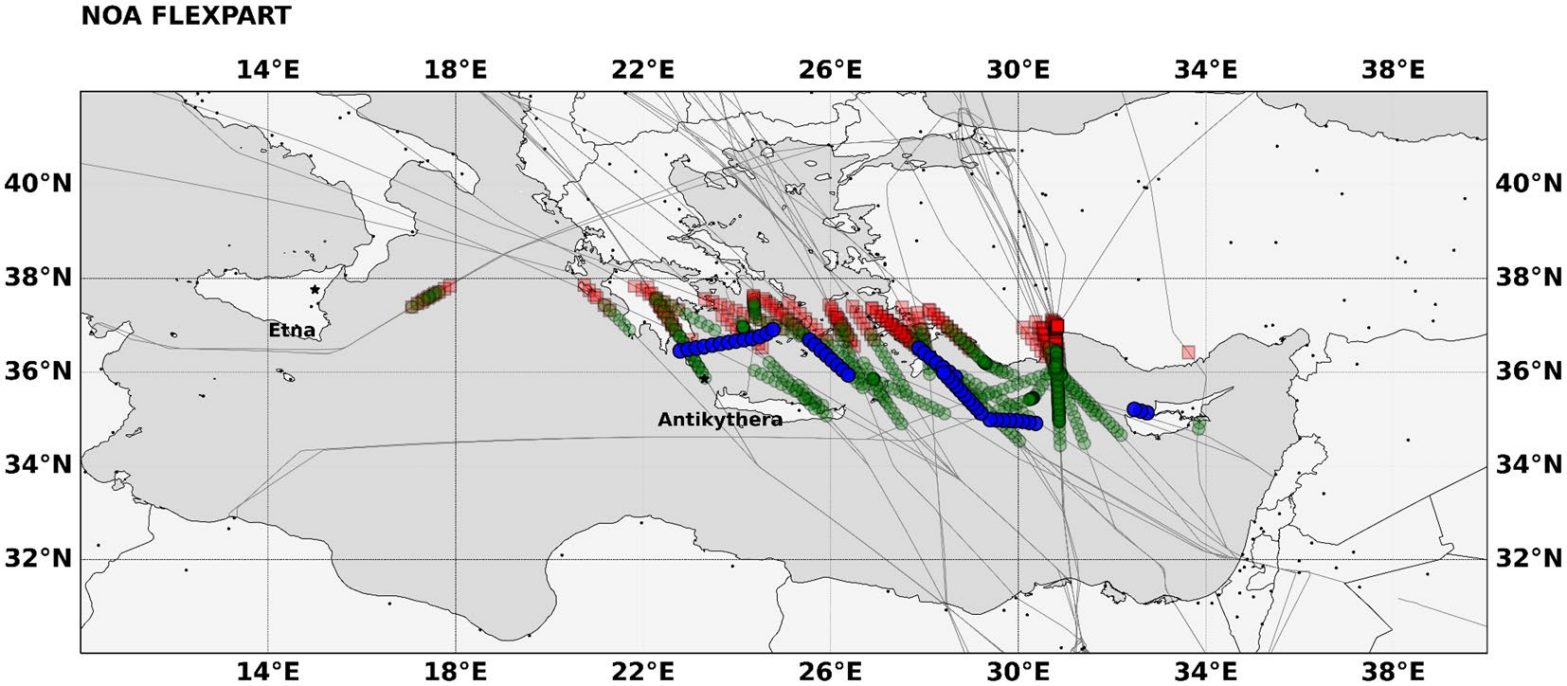
Flights crossed the volcanic plume from 12 March 2021 (08:00 UTC) to 13 March 2021 (08:00 UTC). Green circles indicate flight segments through the volcanic ash plume “w” Aeolus assimilation, whereas red rectangles indicate flight segments through the volcanic ash plume “w/o” Aeolus assimilation. Green and red markers show flight segments for 52 common flights that crossed plumes in both scenarios. Blue solid circles show flight segments of 6 flights that encountered volcanic ash with Aeolus assimilation only. Black lines represent the actual trajectories of the affected/exposed flights. Black dots show numerous airports in the area.

Beyond aviation safety, Aeolus application has the potential to provide improved estimates of the accumulated ash dose for an aircraft crossing the volcanic plume^46^, incurring long term economic damages associated with reductions in engine lifetime or more frequent services. In the extreme case, the ash dose from an eruption might not be considered at all by erroneously assuming that the flight never encountered the ash cloud. To quantify the exposure of air traffic to the volcanic ash cloud and its sensitivity to the Aeolus wind fields, we identify 58 flights that encountered non-zero ash concentrations in the “w” Aeolus simulation along its cruising altitudes from 12 March 08:00 UTC to 13 March 08:00 UTC. Of those flights, 52 have also encountered non-zero ash concentrations in the “w/o” Aeolus simulation, but at different time and location, as shown in Fig. 8. As expected by our simulations, flights encounter the plume erroneously further northward in the “w/o” Aeolus simulation (red squares in Fig. 8). Even though the maximum ash concentration encountered by the 52 flights is well below the recommended threshold, the percentage differences between the “w” Aeolus and “w/o” Aeolus are non-negligible, with magnitudes in the forecasted concentration exceeding 80% in at least 12 flights. Likewise, the accumulated ash dose never exceeds the 40 mgs/m^3^, far below the threshold dosage of 14,400 mgs/m^3^^46^. However, we find significant differences in the exposed doses between the two runs. We emphasize that 6 flights (blue circles in Fig. 8) encountered the volcanic cloud in the “w” Aeolus run only. This implies a 10% false-positive error on the detection of the number of flights encountered the ash cloud in the “w/o” Aeolus simulation, because those flights would have been ignorant to the presence of the volcanic ash plume under the “w/o” Aeolus forecast. Considering the flight restrictions at the time of the eruption because of the pandemic dispersal containment measures, this erroneous attribution is expected to be higher under normal flight conditions. Despite that the Etna eruption of March 12th had low risk for aviation safety, we suggest that the assimilation of Aeolus wind data would provide a more accurate assessment of the air traffic exposure in the case of higher ash concentration levels from a future eruption.

## Summary and conclusion

The benefit of Aeolus wind assimilation for global NWP models has been demonstrated in Ref.^22^, showing significantly improved representations of the wind fields, especially at the Tropics and the Southern Hemisphere. Similar, or even enhanced improvements on wind forecasts are expected when Aeolus wind assimilation is acknowledged in regional NWP models, as shown in the case study presented here. The improvements from Aeolus assimilation are usually observed over under-sampled geographical regions, such as the Mediterranean Sea for our case. Furthermore, upper tropospheric heights seem to benefit more from Aeolus assimilation than the lower troposphere, which could also be related to under-sampling issues (e.g., radiosondes used for data assimilation have worse spatial coverage in the free troposphere).

For all the above reasons, volcanic ash early warning systems can benefit from Aeolus assimilated fields. Volcanoes are mostly located in remote regions that are not covered by surface-based networks of observations. Furthermore, volcanic ash plumes are usually injected at upper-tropospheric and lower-stratospheric heights, and as such, their transport is driven by upper tropospheric winds. In our study we show that for all the above reasons, when we acknowledge Aeolus assimilated wind fields, we reveal improvements on the volcanic ash plume simulation related to its transport, horizontal plume extent, and vertical distribution. These improvements are shown to be important for informed decision-making by the aviation sector, towards improving preparedness and response to hazards arising from volcanic eruptions, related to both aviation safety and economic impacts related to engine damages and maintenance.

Aeolus can benefit similar applications related to aerosol transport and deposition simulations beyond the volcanic ash case, including for example desert dust, sea salt and stratospheric aerosols. The high value of Aeolus for aerosol studies stems from the fact that the satellite provides synergistic wind and aerosol observations over remote areas, such as deserts, oceans, and volcanic arcs, which are mostly under-sampled. As such, the discussion over the potential launch of follow-on Aeolus missions by ESA and EUMETSAT is critical to positively conclude, along with upgrades of the Aeolus lidar specifications in terms of vertical and temporal resolution, and the inclusion of a cross-polarization channel to improve the detection of non-spherical depolarizing aerosol and cloud targets (e.g., desert dust, volcanic ash and ice crystals).

The effect of the Aeolus assimilation in the dispersion of the volcanic particles should be also examined in other cases studies in different regions and with different wind field patterns. Our study is crucial to demonstrate that more wind profiling datasets are needed in the future for improving volcanic ash forecasts.

## Methods

### WRF-ARW and FLEXPART models setup

We have developed an Early Warning System tool at the PANGEA-NOA. The system is based on the WRF-ARW and FLEXPART models, operating in this study on deterministic forecasting mode, although a configuration that exploits ensemble forecasting is also possible^47^. The Advanced Research WRF model version 4^10^ is used to perform meteorological simulations over the study region. The spatial resolution of the model was 12 × 12 km for a total of 351 × 252 grid points, and 31 vertical levels (up to 50 hPa). The simulation period starts at 12 March 2021, 00:00 UTC (six hours earlier than the FLEXPART runs, to accommodate for the model’s spin-up) and ends on 14 March 2021, 18:00 UTC, with hourly outputs.

Table 1 summarizes the Physics Parameterizations (PP) schemes for the WRF-ARW simulations. The initial and boundary conditions for the WRF-ARW runs are produced from the European Centre for Medium Range Weather Forecasts (ECMWF) Integrated Forecast System (IFS), at 0.125° × 0.125° spatial resolution, 137 vertical model levels. The boundary conditions are refreshed at 6-h intervals. Two versions of the IFS^38^ initial condition fields were utilized, one in which Aeolus Rayleigh-clear and Mie cloudy HLOS L2B wind profiles have been assimilated (“w” Aeolus experiment), and one without Aeolus data (“w/o” Aeolus experiment). The initial conditions w/o Aeolus assimilation adopt the model setup utilized in the Observing System Experiment (OSEs) performed by Ref.^29^. Sea Surface Temperature (SST) analysis data were provided by the Copernicus Marine Environment Monitoring Service (CMEMS) at a spatial resolution of 1/12°.

**Table 1:** Configuration of the PP schemes for the WRF-ARW simulations.

PP	Schemes	Refs
Microphysics (MP)	Thompson	^64^
Surface layer (SFL)	Monin–Obukhov (Janjic Eta)	^65^
Planetary boundary layer (PBL)	Mellor–Yamada–Janjic (MYJ)	^66^
Cumulus parameterization (CUM)	Tiedtke	^67^
Longwave and shortwave radiation (RAD)	Rapid radiative transfer model (RRTMG)	^68^
Land surface (LSM)	NOAH	^69^

WRF-ARW outputs, produced by the two different initial and boundary conditions data sets (“w” and “w/o” Aeolus), are compared for assessing the potential improvements attributed to the assimilation of Aeolus wind profiles. The transport of volcanic ash plume was simulated with the Lagrangian particle dispersion model FLEXPART^9,48,49^ in a forward mode. The ash dispersion simulations were driven by hourly meteorological fields from the WRF-ARW model initiated with control and assimilated datasets to quantitatively assess the impact of data assimilation. The use of 1-hourly WRF meteorological fields at a 12 × 12 km spatial resolution allow a more detailed representation of the volcanic plume dispersion. Simulations were initiated at the reported start time of the eruption 07:15 UTC on 12 March 2021 and were completed at 00:00 UTC on 14 March 2021 with a total of 50,000 particles to be released in each forecast. We estimate the mass eruption rate (MER) for ash particles following^37^, by inverting the observed plume height using the 1-D plume model of Ref.^50^. The initial injection height in the model is set to the surface of the Etna summit craters (i.e., 3.3 km a.s.l. up to 9 km a.s.l., based on the VONA reports^36,37^ and field observations). Also, the gravitational particle settling^51^ was determined assuming spherical particles with a density of 2450 kg/m^3^. The size distribution of volcanic ash particles was described using four size bins (5, 9, 13, and 21 µm diameter), as these cover the size distribution relevant for long-range transport (≤ 25 µm diameter).

### PANGEA observatory

The observatory of PANGEA (PANhellenic GEophysical observatory of Antikythera) established its first operations in June 2018. The atmospheric circulation pattern at PANGEA location favours the transport of air masses carrying an abundance of different aerosol types such as windblown Sahara dust, Etna volcanic aerosols or anthropogenic pollution from major megacities. Hence, this coastal site constitutes an ideal place to study natural aerosols under the prevailing background conditions of the Eastern Mediterranean.

Currently, a lidar system the type of Polly^XT^^39,52^ and a CIMEL Electronique sun/sky-photometer^53–55^ operate continuously at PANGEA to provide profiles and columnar aerosol properties with high accuracy and resolution.

Polly^XT^ is a multi-wavelength, Raman, polarization lidar with 24/7 remote operation capability. The system operates in 355, 532 and 1064 nm and is equipped with 12 detectors to measure light elastically and in-elastically (at 387, 407 and 607 nm) backscattered from atmospheric constituents. Polarization capability also enables the detection and vertical separation of non-spherical (e.g., volcanic ash, dust) from spherical aerosols (e.g., smoke, pollution, marine particles).

The CIMEL sun/sky-photometer measures direct solar irradiance and sky radiance at several wavelengths (340, 380, 440, 500, 675, 870, 1020 and 1640 nm), to derive columnar aerosol optical and microphysical properties^56^.

Observations from both sensors are of strong interest for observing Pan-European networks such as the Aerosol, Clouds and Trace Gases Research Infrastructure (ACTRIS-RI), the European Aerosol Research Lidar Network (EARLINET) and the global AErosol RObotic NETwork (AERONET) (https://aeronet.gsfc.nasa.gov/); in all of which measurements taken at PANGEA are submitted on a regular basis.

### Ash mass calculation

Volcanic ash mass estimates were derived from a combination of Polly^XT^ lidar measurements and sun-photometer observations. First, the lidar measurements were averaged over the 2.5-h period when the volcanic layer was observed above Antikythera, and the standardized European Aerosol Research Lidar Network (EARLINET) algorithm (Single Calculus Chain—SCC^57^ was used to derive particle backscatter coefficient (${\beta }_{p}$) and particle linear depolarization ratio (${\delta }_{p}$) profiles.

These profiles were then used to calculate the ash mass concentration with the “POlarization-LIdar PHOtometer Networking” method POLIPHON^43,44^, tailored for Etna ash as described in Ref.^58^.

More specifically, the following equation was used:

1 $${m}_{a}={\rho }_{a}{\times c}_{v,a}\left(\lambda \right)\times {\beta }_{p,a}\left(h,\lambda \right)\times {S}_{p,a}\left(h,\lambda \right),$$

where $m$ is the mass concentration, $a$ indicates an aerosol type, h is the height (a.s.l.) of the lidar measurements, $\rho$ represents the particle mass density, λ is the wavelength, ${c}_{v}\left(\lambda \right)$ is the so-called volume-to-extinction conversion factor, derived from sun-photometer measurements and ${S}_{p}\left(\lambda , h\right)$ is the ratio of the particle extinction to particle backscatter coefficient (lidar ratio).

As ${m}_{a}$ calculation is sensitive to the aerosol type, under simultaneous presence of multiple aerosol components a decomposition of the total aerosol backscatter coefficient ${\beta }_{p, }$ is needed prior to the mass concentration calculation. In POLIPHON, this decomposition is supported for up to two aerosol types, one exhibiting large particle depolarization ratio values (usually dust or volcanic ash) and one that does not (marine, continental or tropospheric smoke and their mixtures).

Polly^XT^ lidar signals are sensitive to aerosol particles in the radius range from about 50 nm to a few micrometers^59^. For FLEXPART, the size range of volcanic ash particles is between 5 and 21 µm diameter, which is within a range that is detectable from Polly^XT^. The POLIPHON technique for the retrieval of the concentrations has been validated against synergistic retrievals that combine multi-wavelength lidar and sun/sky-radiometer observations (sensitive up to 15 μm in particle radius^60,61^) for dust and volcanic ash particles and has been found to perform well^62,63^. To separate the contribution of the depolarizing (${\beta }_{p.d}\left(h, \lambda \right))$ and the non-depolarizing (${\beta }_{p.nd}\left(h, \lambda \right))$ component to the particle backscatter, we apply the following equations:

2 $${\beta }_{p,d}\left(h, \lambda \right)={\beta }_{p}\left(h, \lambda \right)\frac{\left({\delta }_{p}\left(h,\lambda \right)-{\delta }_{p,nd}\left(h,\lambda \right)\right)\left(1+{\delta }_{p,d}\left(h,\lambda \right)\right)}{\left({\delta }_{p,d}\left(h,\lambda \right)-{\delta }_{p,nd}\left(h,\lambda \right)\right)\left(1+{\delta }_{p}\left(h,\lambda \right)\right)},$$

3 $${\beta }_{p,nd}\left(h, \lambda \right)={\beta }_{p}\left(h, \lambda \right)-{\beta }_{p,d}\left(h, \lambda \right).$$

In Table 2, we summarize the values and uncertainties of parameters used as input in the above. The resulting ${m}_{a}$ uncertainty rises from the input parameters’ errors that propagate into the ${m}_{a}$ calculation and is also listed in Table 2.

**Table 2:** Parameters used for lidar profiles decomposition and mass concentration calculation.

	ρ_α_ [cm^−3^]	c_ν,α,532 nm_	δ_p,α,532 nm_ (h)	S_p,α,532 nm_ (h) [sr]
Ash particles	2.6 ± 0.6	0.6 ± 0.1	0.36 ± 0.02	50 ± 10
Sulfates	1.5 ± 0.3	0.18 ± 0.04	0.05 ± 0.01	60 ± 20

### Flight tracking information

We use archived flight data provided by the FlightRadar24.com online database, from which we retrieve tracks for 152 flights crossing Southern Eastern Europe after the Etna eruption between 12 March 08:00 UTC and 13 March 08:00 UTC. Of those flights, we identify the ones that encountered the volcanic ash cloud at the cruising altitudes in both w and w/o Aeolus model simulations. This is possible by linearly interpolating the simulated ash concentration to the time and altitude of the flight track. In this way we identify 58 flights that have encountered non-zero ash concentrations in the “w” Aeolus simulation and 60 flights in the “w/o”-Aeolus simulation. At the end, we identify 52 flights that encounter volcanic ash at cruising altitudes regardless of the scenario.

## Data Availability

The lidar data from the Polly^XT^ at PANGEA station (i.e., attenuated backscatter coefficient and volume linear depolarization ratio), were derived using the Single Calculus Chain (SCC; https://scc.imaa.cnr.it) algorithm; an automatic-analysis tool for lidar data processing, developed within EARLINET (https://www.earlinet.org/) and ACTRIS (https://www.actris.eu/) and are available by the co-author Anna Gialitaki (togialitaki@noa.gr) upon request. Flights data retrieved from www.FlightRadar24.com (online database). The WRF and FLEXPART-WRF models simulation results are also available by the co-author Anna Kampouri (akampouri@noa.gr) upon request.

## Code availability

The WRF model code is publicly available, has a digital object identifier 10.5065/D6MK6B4K and can be obtained via GitHub (https://github.com/wrf-model/WRF). The FLEXPART-WRF model code is publicly available and can be obtained from https://www.flexpart.eu/wiki/FpOthermetinput. The code used for data processing was written with Python programming language version 3.8 (https://www.python.org/) and can be obtained via GitHub: (https://github.com/NOA-ReACT/Aeolus_Volcano_2023). The retrievals and the aerosol lidar optical properties are available from the co-author Anna Gialitaki (togialitaki@noa.gr) upon request.
